# The unmet needs for modern family planning methods among postpartum women in Sub-Saharan Africa: a systematic review of the literature

**DOI:** 10.1186/s12978-021-01089-9

**Published:** 2021-02-10

**Authors:** Jumaine Gahungu, Mariam Vahdaninia, Pramod R. Regmi

**Affiliations:** 1 Martin Luther King University, Bujumbura, Burundi; 2 Peninsula Medical School, Faculty of Health, University of Plymouth, Plymouth, UK; 3 Faculty of Health and Social Sciences, Bournemouth University, Bournemouth Gateway Building, 10 St Paul’s Ln, BH8 8AJ Bournemouth, UK

**Keywords:** Family planning, Contraception, Unmet need, Modern contraception methods, Postpartum, Women of reproductive age

## Abstract

**Background:**

Sub-Saharan Africa has the highest fertility rate in the world, with the highest unmet need for family planning (FP). Yet, there is a lack of knowledge about the determinants for non-utilisation of modern contraceptive methods among women of reproductive age. This systematic review of literature assessed factors affecting the unmet need and reasons for non-utilisation of modern contraceptive methods during the postpartum period in Sub-Saharan African women.

**Methods:**

An online literature search was conducted in several databases: MEDLINE, Cochrane Review, PubMed, Elsevier's Science Direct and Web of Science. The search was completed by hand searching. Data were extracted and summarised using the Arksey and O’Malley methodology.

**Results:**

In total, 19 studies were included; one qualitative study, seventeen quantitative, and one used a mixed-methods approach. Studies were conducted in Ethiopia (n = 11), Nigeria (n = 3), Kenya (n = 2), Malawi (n = 2) and Uganda (n = 1). Factors affecting the unmet need for modern contraceptive methods were described at three levels: (a) individual; (b) household; and (c) healthcare facility level. Reasons for non-use of FP included: fear of side effects; husband’s disapproval; the absence of menses; abstinence; and low perception of risk of pregnancy.

**Conclusion:**

Unmet needs in postpartum FP in women from Sub-Saharan Africa were associated with health-system and socio-demographic determinants. We suggest that there is a need to improve the awareness of modern contraceptive methods through effective interventions. Further research is needed for under-studied countries in this continent.

## Background

Available evidence has shown that short birth spacing puts the mother and both the newborn and the preceding child at high risk of morbidity and mortality [1]. It is gradually being recognised there is a high unmet need for postpartum family planning (PPFP); and that relying on breastfeeding alone may expose women to the risk of unwanted pregnancies [2, 3]. Sub-Saharan African (SSA) countries have the highest unmet need for PPFP [4], whilst there is a recognised knowledge gap in PPFP in SSA countries [5].

Women with unmet need for PPFP can be defined as all sexually active and fecund women (legally married or in a consensual union) wishing to prevent unintended or closely-spaced pregnancies during the first twelve months following delivery but are not using any contraceptive method [6, 7].

Many women in SSA countries know the important role played by Family Planning (FP) in preventing unwanted pregnancies, but what they may not know is its role in planning and improving the lives and families of the users [8]. In developing countries, it was estimated in 2012 that 222 million women had an unmet need for FP [9]. Moreover, reducing the unmet need for FP in SSA and other developing countries would avert more than one million infant deaths and 54 million unwanted pregnancies which, if not prevented, would result in 21 million inadvertent births, seven million miscarriages, and 26 million abortions of which 15 million would be unsafe [9].

By preventing undesirable pregnancy, FP averts maternal and childhood deaths and helps a woman decide freely and conscientiously about her pregnancy spacing and parity [1]. Additionally, when the unmet contraceptive need is reduced, women’s wellbeing, education and autonomy are improved, and the need for unsafe abortion is reduced [10]

Currently, the fertility rate in Africa stands at 4.7 children per woman, which is the highest in the world [11]. One in 26 adult women is at risk of maternal death in SSA due to poor reproductive health, compared to 1 in 7300 women in developed countries [8]. Providing postpartum women with access to FP would thus improve their reproductive health and save them from maternal deaths.

Data from seventeen developing countries have shown that the unmet need for PPFP has reached 88% in some SSA countries and that women in the postpartum period have more unmet needs for modern contraceptive methods than any other women [12, 13]. This is likely to be associated with the lack of FP counselling during the antenatal and postnatal period, and negligence of the PPFP needs by national family planning programmes [14, 15]. It should be noted that four of the six countries with the highest unmet need for FP in the world are in SSA, namely the Democratic Republic of the Congo (DRC), Uganda, Nigeria, and Kenya [16].

Conversely, contraceptive uptake within a short time after the childbirth was found to increase contraceptive discontinuation, which consequently put postpartum women at higher risk of pregnancy. For example, in some countries in SSA, South Asia, Asia Pacific, and South America early users of modern contraceptives experienced higher pregnancy risks than non-users due to the discontinuation of the contraceptives around the same time they are at most need [16, 17]. Among women who discontinue one modern contraceptive method due to its side-effects or other problems related to the method, only 13 percent switch to another method [18]. This would suggest that there is a need to understand these deep-rooted reasons for the lack of uptake of PPFP as these might go beyond the non-availability of modern contraceptives in health facilities or the economic situation of SSA countries. For this reason, the current review assessed both the factors associated with women’s unmet need for PPFP and their reasons for not utilising contraceptives.

Although extensive research has been carried out on the unmet need for PPFP, no single review investigating both unmet need for PPFP and the reasons for non-use in SSA exists. Earlier reviews on FP omitted to study the unmet need in the postpartum period [4, 19]. Other studies [7, 20] have also considered reasons for non-use of FP methods but focused neither on postpartum women nor SSA countries. Therefore, the focus of this review is unique which has aimed to address two questions about PPFP need in SSA: (a) investigate the unmet need for modern FP; (b) identify associated factors and reasons which lead women not to utilise modern FP in SSA.

## Methods

Studies that met the inclusion criteria detailed below were included.

### Inclusion and exclusion criteria

To be included, studies had to (i) examine and identify factors affecting the unmet need for modern FP, (ii) include modern contraceptive methods, (iii) to access current research in this area, published between January 2014 and December 2019 [21], (iv) report specific results only in SSA countries, (v) included only women in their twelve-month postpartum period, (vi) have accessible full-length articles, (vii) published in the English language, and (viii) were peer-reviewed and of any study design (qualitative, quantitative, or mixed methods) and the latter was decided due to the complexity of topic [22]. Exclusion criteria were the direct opposite of these inclusion criteria. Additionally, studies examining factors associated with only one modern contraceptive method were excluded.

### Search strategy

**Fig. 1:**
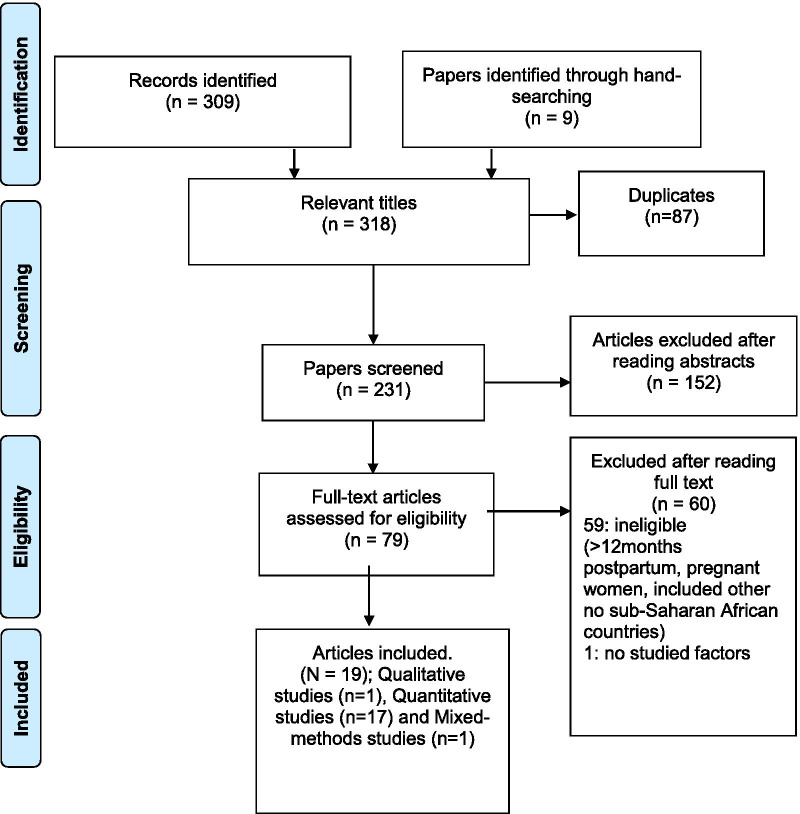
PRISMA flow diagram

The PICO framework [23–26] was author-adapted (PIO: Population: postpartum woman, Intervention: unmet need for modern FP methods and Factors as Outcomes) to formulate the research question. Though this framework helped formulate and hone the research question, this tool was only one contributing factor. The review followed the framework for reviews by Arksey and O’Malley [27] which is a standard approach for conducting scoping reviews, comprising five stages: identifying the research question, identifying relevant studies, study selection, charting the data and collating, summarising and reporting the results. PRISMA checklist [27, 28] was used, and a flow diagram (see Fig. 1) is presented to explain different phases of the article selection as described in the PRISMA guidelines [29].

To evaluate factors affecting the unmet need for PPFP in SSA and summarise evidence for practice [30], a literature review of recent studies was carried out. An initial pool of articles was built by searching several bibliographic databases in a UK-based University’s Library e-resources: MEDLINE, Cochrane Review, PubMed, BMC Health Services Research, Elsevier's Science Direct and Web of Science. Additional articles were retrieved through hand-searching (e.g., visiting the organisation’s websites) and reference mining (i.e., consulting reference list of relevant sources to explore other related literature [31, 32]. Furthermore, PROSPERO was searched for a broader range of results to check whether a similar review is registered [33]. To develop a more inclusive search, Google Scholar was searched; however, with a degree of prudence as it covers non-empirical, and often irrelevant, evidence.

The following combination of keywords and their synonyms was developed and applied in the search, *population*: postpartum women, postpartum, puerperium, postnatal, perinatal; *Intervention*: (a) family planning, birth spacing, contraceptive method, birth control, contraception, birth limiting; (b) unmet need, gap, non-use, unmet trend; *outcome*: factor, challenge, barrier, predictor, cause, reason. Wildcard characters were used to include plurals or for words that may have more than one spelling [34].

For each bibliographic database, appropriate syntaxes with field codes, Boolean operators, and parentheses were used. It is commonly known that each database follows a set of rules and symbols to operate effectively, but at the same time, that some syntax rules are similar for most databases [35]. Therefore, appropriate syntaxes that take into account all similarities and differences were formed and faithfully employed. However, due to the paucity of studies on this topic in SSA [5], the use of Boolean “AND” alone was reduced when searching for articles to include in this study as it could have highly limited the number of retrieved papers, with the risk of excluding eligible studies [36]. As an alternative, the specifier “OR” was added to find as many potential articles as possible on postpartum FP in SSA and based on the eligibility, the list was afterwards narrowed down.

### Screening

Included studied were first assessed against their title and abstracts and the full texts were afterwards screened [34, 36]. To prevent any eventual location, researcher, and publication bias [37], papers that met the eligibility criteria were identified and then independently reviewed by two reviewers. Any disagreements were discussed in detail between all three authors. Duplicates were automatically removed and where relevant, manually removed [34].

### Data extraction

Nineteen studies were assessed and confirmed by two different reviewers. The following information was extracted from the included papers: authors; country and setting, sample, recruitment period, methods and summary points, and key findings. The summary points and key findings included study highlights, factors at the multivariate level, and reasons for not using PPFP.

### Quality appraisal

This review primarily aimed to collect available evidence that leads postpartum women not to use modern contraception and to provide a description of the current state of knowledge on the topic. For the quality appraisal of all the 19 studies, an auto-adapted checklist from CASP qualitative checklist [38], Moule et al. [39], and a checklist used in similar systematic reviews [40] was made and used as shown in Appendix 1 (summary of quality-assessment criteria).

Each element of quality-assessment was scored on a binary level, with 1 implying that the criteria were met and 0 that it was not. Each study was assessed based on the following nine criteria: research aim; defined demand; research design; sampling/recruitment; data collection; methods; data analysis; ethical consideration; reliability. A study was considered high-quality if the sum of met criteria is six or higher and low-quality if its score is five or lower. However, due to the small number of eligible studies, no study was excluded based on quality.

Ethical approval for a literature review is not required [32], it was not thus sought.

## Results

The search of databases and hand-searching provided a total of 318 papers, after adjusting for duplicates, 231 papers remained. After reading abstracts, 152 papers were excluded. The full text of 79 articles were assessed for the inclusion criteria, and nineteen studies met those criteria (see Fig. 1). Of these, eleven were conducted in Ethiopia [41–50, 59], three in Nigeria [51–53], two in Malawi [54, 55], two in Kenya [56, 57] and, one in Uganda [58].

Seventeen studies used quantitative methods, one mixed-methods, and one used a qualitative approach (Table 1). Of the studies using quantitative/mixed-methods, only one was prospective cohort research [51], the remainder followed a cross-sectional design (see Table 1). Twelve studies (63.2%) recruited participants from the community-based settings and seven (36.8%) from health institutions. Most studies (63.2%) were published between 2018 and 2019. It should be noted that expanding the publication period for studies on unmet need for PPFP up to 10 years did not increase the numbers of relevant studies included.

**Table 1:** Characteristics of the included studies

Authors	Country and state/region	Setting	Recruitment period	Type of study/method	Sample size
Abera et al. 2015 [41]	Ethiopia, Gondar	Community-based	August 2013	Cross-sectional/quantitative	N = 703
Abraha et al. 2017 [42]	Ethiopia, Aksum	Community-based	March–April, 2015	Cross-sectional/quantitative	N = 590 (N expected [NE] = 601)
Abraha et al. 2018 [43]	Ethiopia, Aksum	Community-based	March–April 2015	Cross-sectional/ quantitative	N = 590 (NE = 604)
Achwoka et al. 2018 [56]	Kenya	Nationally representative	2013 (month not specified)	National cross-sectional/quantitative	N = 955 (NE = 1012)
Gebremedhin et al. 2018 [44]	Ethiopia, Addis Ababa	Community-based	May–June 2015	Cross-sectional/quantitative	N = 803 (NE = 849)
Dona et al. 2018 [45]	Ethiopia, Aroressa	Community-based	March–April, 2017	Cross sectional/quantitative	N = 684 (NE = 695)
Berta et al. 2018[46]	Ethiopia, Gondar	Healthcare facility	March–April 2015	Cross-sectional/quantitative	N = 404
Bwazi et al. 2014 [54]	Malawi, Ntchisi	Healthcare facility	July 2011	Cross-sectional/quantitative	N = 383
Thindwa et al. 2019[55]	Malawi	nationally representative (nested)	May 2015–May 2016	Cross-sectional/quantitative	N = 578
Chinaeke. 2019 [51]	Nigeria (the Federal Capital Territory and Nasarawa state)	Healthcare facility	April 2014–September 2015	Prospective cohort study/quantitative	N = 399 (NE = 497)
Embafrash and Mekonnen 2019 [59]	Ethiopia, Tahtay-Koraro	Healthcare facility	February–March 2014	Cross-sectional/mixed methods	N = 409 (NE = 422)
Idowu et al. 2015 [52]	Nigeria, Ogbomoso	Healthcare facility	Three months, year not specified	Cross-sectional/ quantitative	N = 444
Iliyasu et al. 2018 [53]	Nigeria, Kano	Healthcare facility	January–February 2015	Cross-sectional/ quantitative	N = 317 (NE = 371)
Keesara et al. 2018 [57]	Kenya, Nairobi	Healthcare facility	December 2013–April 2014	Cross-sectional/qualitative	N = 91
Tegegn et al. 2017 [47]	Ethiopia, Dessie	Community-based	December 2014	Cross-sectional/quantitative	N = 383
Mengesha et al. 2015 [48]	Ethiopia, Dabat	Community-based	January 2013	Cross-sectional/ quantitative	N = 899 (NE = 816)
Gejo et al. 2019 [49]	Ethiopia, Hossana	Healthcare facility	June–July 2018	Cross-sectional/ quantitative	N = 368
Sileo et al. 2015 [58]	Uganda, Butambala	Healthcare facility	2010, month no specified	Cross-sectional/ quantitative	N = 258 (NE = 301)
Abraha et al. 2018 [50]	Ethiopia (Tanquae-Abrgelle, Adwa, Tahtay-Maychew and Laelay-Maychew)	Community-based	March–April 2017	Cross-sectional/ quantitative	N = 1109

*NE*  number expected

The studies by Abraha 2017 [42] and 2018 [43] were conducted on the same sample; however, the authors have reported different results in the two published papers. Therefore, we decided to keep both papers in this review. Table 1 shows the characteristics of the included studies in the review.

The main findings of the included studies are presented in Table 2 and whether stated, also show the main reasons for not using the PPFP (10 studies in total). Highlights of the included studies indicate that many women did not visit the postnatal care services (PNC) and that the need for PPFP services as stated in most of the studies (Table 2).

**Table 2:** Main findings of the included studies

Authors	Findings (multivariate analysis)	Highlights of the study	Reasons for non-use of FP (if stated)
Abera et al. 2015 [41]	Age (CI: 1.04–6.04, p < 0.001) Resumption of menses (CI:5.85–14.63; p < 0.0001) Postpartum period (CI: 2.51–9.30; p = 0.034) Antenatal care* service (CI: 2.57–17.00; p = 0.001) Postnatal care** service (CI: 1.01–2.61; p = 0.042)	97.15% of postpartum women had a good intention about PPFP (51.1% for spacing and 46.1% for limiting) 71.4% had resumed sexual activity Postpartum Contraceptive use (48.4%)	Low perceived risk of pregnancy (49.0%) Husband not living at home (16.8%) Medical problem (11.6%) Fear of side effect (7.7%) Spousal disapproval (6.3%) Religion (4.7%)
Abraha et al. 2017 [42]	Women’s education (secondary, CI:1.29–14.00; p = 0.02 and tertiary, CI:1.14–25.45; p = 0.03) Menses resumption (CI:3.14–13.39; p < 0.001) Problems with previous contraceptive use (CI:0.16–0.72; p = 0.05) Resumption of sexual activity (CI:3.74–24.27; p < 0.001) Prenatal and postnatal FP counselling (CI:2.67–12.28; p < 0.001) PNC service (CI:1.15–4.87; p = 0.02)	48% of postpartum women used modern contraceptives 28.9% had resumed sexual activity at 3-week postpartum	Absence of menses (65.7%) Fear of side effect (11.10%) A single mother (7.80%) Spousal disapproval (7.20%)
Abraha et al. 2018 [43]	Knowledge of modern contraceptives (CI: 1.69, 15.82) Postpartum resumption of sexual activity (CI: 1.34, 3.92) Husband's approval of FP (CI: 2.02, 5.57)	84.3% of postpartum women intended to utilise modern contraceptives, of which 83.3% were for spacing and 6.7% for limiting Only 43.7% of respondents attended PNC, that percentage was 98.1% for ANC	Not stated
Achwoka et al. 2018 [56]	Age (CI: 1.00–1.61; p = 0.05) Women's education (CI:1.01–1.04; p = 0.004) Assisted delivery in a health facility (CI:1.06–1.49; p = 0.008) PF discussion during PNC (CI: 1.10–1.42; p = 0.001) PF discussion during ANC/ PNC (CI: 1.18–1.51; p < 0.001)	77% of the participants needed PPFP, 30% of whom were not currently using any modern FP Only 63% of last pregnancies were desired	Not stated
Gebremedhin et al. 2018 [44]	Marriage (CI: 0.03–0.22) Menses resumption (CI: 1.37–3.41) Postpartum period (CI: 1.18–4.75) History of previous PF use (CI: 0.07–0.18)	39.10% of participants did not know the number of children they wish to have 54.5% were not counselled about FP during their ANC	Not stated
Dona et al. 2018 [45]	Return of menses (CI: 1.47–3.81) FP communication with husband (CI: 1.09–2.41) ANC (CI: 1.23—3.01) PNC (CI: 1.23- 2.94)	Postpartum contraceptive use was at 31.7% 68.7% of participants delivered their last birth at home	Resumption of menses (46%) Distance to the health facilities (38%) Husband’s disapproval (19%) Lack of their preferred methods (11%)
Berta et al. 2018 [46]	Resumption of menses (CI: 2.33–6.35) Resumption of sex (CI: 1.80–5.58) Postpartum period (CI: 1.11–5.55) Knowledge on FP (CI: 2.23–11.24) Husband’s approval (CI: 1.16–3.82)	Participants’ reproductive intention was for 41.8% for spacing and 33.7% for limiting	Non-menstruating (30.9%) Side effects (11.1%) Husband's disapproval (10.6%) Breastfeeding (14.3%)
Bwazi et al. 2014 [54]	Women's education (p = 0.004) Age (p = 0.050) Side effects (p = 0.001) Knowledge of the PPFP services (p < 0.001) Duration of lactation amenorrhoea (p < 0.001) Sexual activity resumption (p < 0.001) Desired number of children (p = 0.020) Number of children (Primiparous) (p < 0.001) Clarity of FP information (p = 0.014) Husband's approval (p < 0.001) Husband’s assistance (p < 0.001) Spousal discussion on FP (p < 0.001) Counselling on FP (p = 0.026)	23% of women had five or more children 38% of women and 33% of their husbands wanted five or more children 22.2% had at least one abortion	Menstruating women (11.40%) Unwilling to use FP (5.20%) Fear of infant death (22%) A single mother (3%)
Thindwa et al. 2019 [55]	Age (CI: 1.8–9.9) Parity (CI: 1.8–39.5) Partner of unknown HIV-status (CI: 1.2–4.0)	41.8% of respondents had an unplanned index pregnancy among whom 35% had an unmet need for FP, and 65% had a contraceptive failure 49.6% of women who did not desire any future child were not using contraception	Not stated
Chinaeke et al. 2019 [51]	Disclosure of women’s HIV status to their partner/relative (CI: 1.2–3.3; p = 0.01) Mentor Mother (CI:0.3–0.8; p < 00.01) FP counselling (CI: 1.1–4.8; p = 0.03)	87.5% of the postpartum women had received FP counselling 49.9% of respondents were not using modern contraceptive methods	Not stated
Embafrash and Mekonnen 2019 [59]	Postpartum period (CI: 4.24–15.71) Low perception of pregnancy risk (CI: 1.04–3.09) Rural residence (CI: 2.57–19.95)	Unmet need for contraception was 36.7% of which spacing was 29.6% and limiting 7.1% 74.8% of respondents had resumed sexual intercourses and 23.7% of them were denied of PF counselling by health providers	Non-menstruating (69.6%) Side effects (13.5%) Abstinence (8.7%) Husband's disapproval (5.2%) Breastfeeding (4.5%) Religion (2.4%) Refusal to remove implants by health providers
Idowu et al. 2015 [52]	Postpartum family planning awareness (CI: 0.0084–0.276; p < 0.001)	46% of respondents had an unmet need for spacing and 56% for limiting	Lack of awareness (17%) Fear of side effects (17.4%) Distance to the health facility (13%) Husband's disapproval (15%) Preferred method not available (13.1%) Low perceived risk of pregnancy (12.7%); Poverty (12.3%)
Iliyasu et al. 2018 [53]	Women’s education (CI:1.03–6.69; p = 0.043) Baby’s age/postpartum period (CI:1.06–3.49; p = 0.031) Resumption of sexual intercourse (CI:0.088–0.38; p = 0.001) Resumption of menses (CI:0.21–0.75; p = 0.004)	67.9% of women resumed sexual activity two months of delivery, of whom 34.4% were not using any modern contraceptive methods 33% were in polygamous unions	Not stated
Keesara et al. 2018 [57]	Qualitative study and the themes are presented	88% of interviewed women were on contraceptive methods by three-months postpartum	Fear of side-effects Husband's disapproval (25%) Fear of infertility
Tegegn et al. 2017 [47]	Knowledge of Lactational Amenorrhea Method (CI: 4.10, 15.02; p = 0.001) Low women's education (CI: 1.22–7.57; p = 0.017) ANC service (CI:1.11–5.79; p = 0.050) PNC service (CI:2.13–6.19; p = 0.0001)	44% of respondents had an unmet need for contraception of which 43% for limiting and 57% for spacing The current infant was unwanted by 7.9% of participants	Low perceived risk of pregnancy (47%) Fear of side effect (16%) Abstinence (9%) Lack of their preferred Method (8%)
Mengesha et al. 2015 [48]	Urban residence (CI: 2.93–11.63) Husband’s education (CI: 1.49–5.97) PNC services (CI: 1.06–4.52) Assisted delivery by health provider (CI: 1.01–3.51)	Postpartum contraception use was 10.3% of which 30.1% was for limiting Home delivery was at 81.1%	Not stated
Gejo et al. 2019 [49]	Women's education (CI: 0.09–0.74) Sex resumption (CI: 1.53–11.52) Menses resumption (CI: 3.07–23.23) Postpartum period (CI: 0.11–0.64)	73.9% of respondents were on contraception (85.29% of them for spacing and 7.72% for limiting)	Absence of menses (32.29%) Absence of spouse (20.8%)
Sileo et al. 2015 [58]	Women’s education (CI: 1.05–3.95; P = 0.04) Prior use of contraceptives (CI: 1.42–83.05; p = 0.02) discussion on contraceptive use with partner (CI: 1.34—2.44; p < 0.001)	74.8% of participants were not using any effective contraceptive method 63.5% reported being victims of emotional abuse and 45.8% of physical abuse	Not stated
Abraha et al. 2018 [50]	Wealth (CI:1.1–3.2; p < 0.001) Partner/husband's education (CI: 1.1–2.6; p < 0.001) PNC service (CI:1.9–4.3; p < 0.001) Distance to the health facility (CI: 2.7–4.6; p < 0.01) ANC service (CI: 1.9–4.2); p < 0.01)	49.3% attended the four ANC visits recommended by WHO 96.90% of respondents and 68.1% of their partners had tested for HIV 13.5% had complications during the puerperium period	Not stated

*ANC*  antenatal care, *PNC* postnatal care

Overall, this review found that generally factors associated with unmet need for PPFP (Tables 2 and 3) can be grouped in three levels as discussed below.

**Table 3:** Major factors affecting the use of PPFP methods in SSA countries at three levels*

Main themes	Number of Studies	Findings (multivariate analysis)
**Individual level**		
Women’s education (6 studies)	7 studies	Decreased effect: Abraha et al. 2017 [42]; Achwoka et al. 2018 [56]; Bwazi et al. 2014 [54]; Iliyasu et al. 2018 [53]; Tegen et al. 2017 [47]; Gejo et al. 2019 [49]; Sileo et al. 2015 [58]
Women’s age	4 studies	Increased effect: Abera et al. 2015 [41]; Achwoka et al. 2018 [56]; Bwazi et al. 2014 [54]; Thindwa et al. 2019[55]
Women’s parity	2 studies	Increased effect: Thindwa et al. 2019[55]; Bwazi et al. 2014 [54]
Resumption of menses	7 studies	Decreased effect: Abera et al., 2015 [41]; Abraha et al., 2018 [43]; Gebremedhin et al., 2018 [44]; Dona et al., 2018 [45]; Berta et al., 2018 [46]; Gejo et al., 2019 [49]; Iliyasu et al., 2018 [53]
Resumption of sexual activity	6 studies	Decreased effect: Abraha et al., 2017 [42]; Abraha et al., 2018 [43]; Berta et al., 2018 [46]; Gejo et al., 2019 [49]; Iliyasu et al., 2018 [53]; Bwazi et al., 2014 [54]
Knowledge of FP methods	5 studies	Increased effect: Abraha et al., 2018 [43]; Berta et al., 2018 [46]; Tegegn et al., 2017 [47]; Idowu et al., 2015 [52]; Bwazi et al., 2014 [54]
**Household level**		
Husband’s approval	3 studies	Increased effect: Abraha et al., 2018 [43]; Berta et al., 2018 [46]; Bwazi et al., 2014 [54]
Husband’s education	2 studies	Increased effect: Mengesha et al., 2015 [48]; Abraha et al., 2018 [50]
FP discussion with husband/partners,	3 studies	Increased effect: Dona et al., 2018 [45]; Bwazi et al., 2014 [54]; Sileo et al., 2015 [58];
**Health care facility level**		
ANC	5 studies	Decreased effect: Abera et al., 2015 [41]; Tegegn et al., 2017 [47]; Dona et al., 2018 [45]; Abraha et al., 2018 [50]; Achwoka et al., 2018 [56]
PNC	7 studies	Increased effect: Abera et al., 2015 [41]; Dona et al., 2018 [45]; Tegegn et al., 2017[47]; Abraha et al., 2018 [50]; Achwoka et al., 2018 [56]; Abraha et al., 2017 [42]; Mengesha et al., 2015 [48]

### Factors influencing the unmet need for PPFP at an individual level

Women’s education was a major factor contributing to their unmet need for PPFP as revealed in seven studies [42, 47, 49, 53, 54, 56, 58]. Other studies showed that the association was found at the bivariate and not at multivariate analysis level [41, 45, 48, 50, 52, 59]. In contrast to the studies mentioned above, women’s education was not a determinant of unmet contraceptive need in two studies [44, 51].

Of the thirteen studies that explored women’s age as a factor of unmet need for PPFP, three studies found it statistically significant at multivariate regression level [41, 55, 56] and five studies at the bivariate level [47, 48, 53, 54, 59]. Additionally, for some studies, the unmet need for FP was lower in younger postpartum women [41, 56] as opposed to [55] who concluded the unmet need to be higher in younger postpartum women.

Women’s parity was found to be a factor influencing the unmet need for PPFP in two of the included studies. Bwazi and colleagues [54] concluded the wanted number of children statistically affects the unmet contraceptive need, whilst Thindwa et al. [55] showed that primiparous women had a greater unmet need for contraception than women with three or more parities. The number of living children was not significant at multivariate regression analysis, but the significance was, however, found in five studies at the bivariate level [41, 42, 48, 53, 56].

Though breastfeeding was given as one of the reasons not to use PPFP [46, 47, 59], only one study found a significant association between the duration of lactation amenorrhoea and the PPFP services [54]. The resumption of menses was associated with PPFP in seven studies [41, 42, 44–46, 49, 53].

Several other factors affecting PPFP in SSA were examined at this level. The resumption of sexual activity was discussed in six studies and was found to positively influence the uptake of PPFP in all [42, 43, 46, 49, 53, 54]. The length of postpartum period, i.e. the age of the infant, influenced the utilisation of PPFP in six studies at multivariate level [41, 44, 46, 49, 53, 59]. The knowledge that the respondents had of FP was examined in ten studies and was found to be negatively associated with unmet need in five studies [43, 46, 47, 52, 54]. Furthermore, prior use of contraceptives, low perceived risk of pregnancy, and women’s wealth/income were also determinants of the PPFP (Table1).

### Factors influencing the unmet need for PPFP at the household level

The husband’s approval was examined in four studies and was a significant factor of unmet need for FP in three of the studies [43, 46, 54]. Other associations with unmet need for PPFP were examined in relatively few studies and found statically significant, which included husband’s education [48, 50], FP discussion with husbands/partners [45, 54, 58], husband’s assistance [54], disclosure of HIV status to husband/partner and mentor mother [51].

### Factors influencing the unmet need for contraception at health care facility level

Among the eight studies that evaluated antenatal care (ANC) services, five found a strong association with the unmet contraceptive need [41, 45, 47, 50, 56]. Those five studies along with two others [42, 48] revealed postnatal care (PNC) services to be determinants of PPFP uptake. The FP counselling provided by health care providers during both ANC and PNC services was positively associated with the uptake of PPFP in four studies out of five that examined this factor [42, 51, 54, 56]. Additionally, though the association was not established with an adjusted odds ratio, crude odds ratio concluded an association between ANC and the unmet contraceptive need in three more studies [42, 48, 49].

Reasons for not utilising PPFP were raised in ten studies. The major reasons reported by most participants were ‘no return of menses’ stated in six studies, the ‘fear of side effects’ mentioned in eight studies, ‘husband’s disapproval’ stated in six studies, ‘abstinence’ and ‘low perceived risk of pregnancy’ respectively mentioned in three and two studies, ‘lack of preferable contraceptive method’ stated in three studies, ‘fear of infertility’ stated in two studies. Other reasons raised by few respondents (less than 4%), sometimes examined in only one study, as showed in Table 2, which included ‘poverty’, ‘health provider incompetency’, ‘unwilling to use family planning’, and ‘fear of death’.

It should be stressed that religion was mentioned in only two studies by less than 5% of the study participants as one of the reasons they were not on modern contraceptive methods [41, 59], which might be explained by the fact that, as suggested by [60], religious women can still prefer to use modern contraceptives despite religious opposition.

**Fig. 2:**
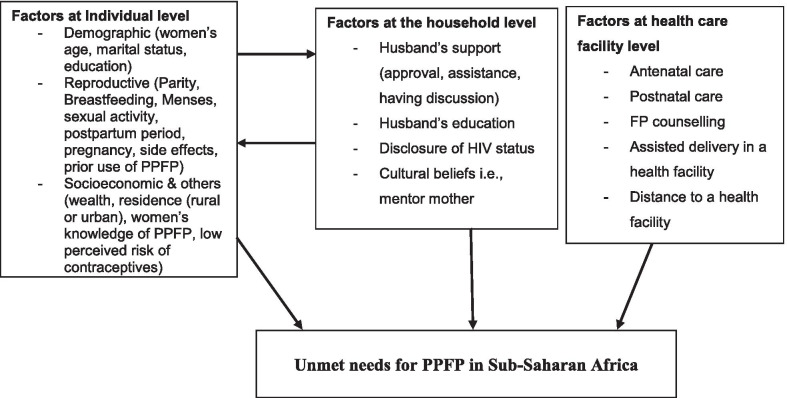
Conceptual diagram

A conceptual diagram was developed presenting a theoretical connection between the individual, household, and health facility characteristics and unmet needs for PPFP in women in SSA countries (Fig. 2).

## Discussion

This is the first study to systematically review both the factors influencing the unmet need for PPFP and the reasons raised by postpartum women on their utilisation of modern contraceptive methods. The results in this review are based on nineteen original studies, published within the last five years which studied determinants of unmet need for PPFP and reasons that lead women in the postpartum period not to use modern contraceptives in SSA. The reviewed articles provided evidence for the unmet contraceptive needs and this review compared and highlighted all the factors and predictors reported in the studies. Reasons for not using contraceptive methods put forward by the postpartum women were mostly different from statistically significant factors affecting the use of contraception.

The low education level of either women or their husbands/partners was found to be a major factor of unmet need for FP in many studies. Evidence suggests that women’s education can contribute to the improvement of contraceptive uptake during the postpartum period and therefore reduce the unmet PPFP need [61–63]. A husbands’ education was a key factor in the promotion of contraceptive use [64]. This might be explained by the fact that the educational status of either partner plays a crucial role in empowering both spouses, reducing gender inequality, and promoting discussion and support within a household. Hence, to be able to increase their utilisation of contraception and thus reduce the unmet need for PPFP, the education of both women and men need to be improved and, health providers should focus more on women with less education when they visit the ANC and PNC services.

The age of women, either young or old, depending on the type of contraceptives, can affect the PPFP as found in this literature review. This converges with the findings of a study conducted in the USA (United States of America) which showed that being younger (age 21–25) was a risk factor for limited contraceptive methods [65]. This might be because, as suggested by young postpartum women, they face more problems accessing PPFP programmes than older ones in SSA. It could be that contraceptive use can be higher in younger women because they are more sexually active, thus tend to utilise more contraceptive methods than their counterparts [66].

The resumption of menses was also associated with the contraceptive use and, ‘breastfeeding’ and ‘no return of menses’ were among the reasons for not using contraceptives in the included studies. Data from seventeen countries showed that the resumption of menses was a determinant of unmet need for PPFP [67] and another multicentre study in Kenya, Peru, the Dominican Republic and Indonesia found an association between breastfeeding and unmet need for PPFP [68]. The possible reason for the association might not only be that most women in Africa assume being at risk of pregnancy when their menses return but also that, healthcare providers state to women that menstruation is a sign of fecundity and hence, advise women to seek for FP services after the resumption of their periods [69]. Additionally, as the majority of women in SSA breastfeed for a relatively extended period [70], they might be reluctant to initiate any modern contraceptive method soon after birth believing there is no risk of pregnancy. Studies have shown that the extended period of breastfeeding for up to two years in SSA can substantially lengthen the period of amenorrhea and that the sexual abstinence can be respected for an extended period after a birth influence, which should generally reduce the unmet need for PPFP [3]. However, the return of fecundity is often unpredictable among women [71, 72]. The longer the postpartum period, the higher the likelihood of ovulation becomes, and ovulation usually precedes the first return of menses [73]. Therefore, adequate knowledge about pregnancy risk should be provided to both women prior to the return of their menstruation and the health providers as part of their continuous training because, as documented, FP counselling is overlooked by some health providers during ANC and PNC [72].

Women in the postpartum who knew about FP were more likely to utilize PPFP than their counterparts. This result was in line with other studies conducted in Africa [74, 75]. This tells us that women’s exposure to FP information can increase demand for FP services and that sub-Saharan countries need to prioritise raising awareness on PPFP. This can cause positive behavioural change [76], addressing unmet FP needs, and help prevent abuse and unsafe abortion [77, 78]. While very little is known in Africa about the latter, the few existing studies suggest high rates of unsafe abortion and that improving FP services would be the best solution to prevent abortion [78, 79], corroborating therefore Bwazi et al. [54] who revealed a high rate of abortion among their respondents.

Findings from this review have also highlighted the influence of partners in using PPFP. The role of spouses’ disapproval and discussing using modern contraceptives with male partners shows how deep the inequity is in the household in terms of decision making. In most, if not all, SSA countries, the husband generally makes all important decisions for the family, including reproductive ones [80]. Sometimes secret contraceptive use is adopted where a woman in a family faces opposition and barriers to FP utilisation. However, FP users mostly respect their husbands’ decisions. This highlights the need for husbands or male partners to be involved in maternal health services and, most importantly, encouraged to accompany their wives/partners to ANC and PNC services as recommended in some Asian countries [81].

ANC and PNC services and ‘FP counselling’ provided by health care providers have been identified as major contributing factors to the unmet need for PPFP methods in the majority of the studies included in this review. Women who did not receive ANC and/or PNC were significantly less likely to adopt any modern contraceptive during their postpartum period. Though ‘the FP counselling’ to pregnant women was not statistically significant in influencing the unmet need for PPFP in studies conducted in Tanzania and Uganda [82, 83], the findings of this review were similar to those reported in order parts of the world, including in SSA [84, 85], Mexico [86] and the USA [60]. Previous studies in SSA have shown that ANC and PNC services are often limited [87, 88] and that most mothers who deliver at home do not visit health facilities afterwards to receive PNC [48]. The integration of FP services into maternal and other child health services such as ANC, PNC, and immunisation services, therefore, should be improved. Our review suggests programmes to reach women who delivered at home should be implemented in all those areas where the rate of home delivery is high. In the majority of studies, postpartum women provided substantive reasons as to why they were not using any modern contraceptive method. Similarly, the same reasons have been reported in many other low- and middle-income countries [16, 89].

### Strengths and limitations

This review included recent studies assessing up-to-date factors and findings that will attract researchers in the field to orient their future research towards PPFP in SSA. Although the selection of studies for this literature was systematic, it is possible that some relevant publications were missed, such as studies published in non-English journals. The included studies were dominated by cross-sectional design which can generally show an association rather than causality and, there were a limited number of countries. Consequently, this might have reduced the generalisability of the findings on the unmet need for PPFP to the entire SSA. However, this review will be of interest to those tasked with the improvement of FP programmes; and health policymakers in Africa. A concern about the interpretation of results was the geographic distribution of the studies, as eligible studies came from only five out of 48 SSA countries [90]. The majority of studies focused on individual-level factors and presented a lack of contextual factors related to the healthcare system. Though reasons for not using modern contraceptives were given in most studies, more qualitative studies complement our findings for the reasons raised by postpartum women. However, the outcomes of this review remain relevant for the whole of Sub-Saharan Africa because of similarities in economic and socio-cultural circumstances and health systems across countries [91, 92].

## Conclusions

This review uncovered several plausible and significant factors that highlight the unmet needs for FP amongst of postpartum women in SSA. Reasons for the limited utilisation of modern contraceptives among women were discussed, and the significant determinants are now foregrounded. There is a clear knowledge gap, and the lack of awareness regarding the use of modern FP methods and their effectiveness during the postpartum period in SSA women has been identified. Further research is needed into the impact on the use of FP by women; intimate partner violence; and the association between abortion and the unmet need for modern contraceptives in SSA. Effective interventions and programmes that address the PPFP needs and managing their side effects are urgently required to reduce the unmet need for modern FP methods for postpartum women in SSA.

## Data Availability

Not applicable.

## Appendix 1. Critical appraisal of the included studies

**Table:**

Authors	Research aim	Defined demand	Research design	Sampling/recruitment	Data collection	Methods	Data analysis	Ethical consider	Reliability	Number of criteria (quality)
Abraha et al. 2018 [43]	✔	✔	✔	✔	✔	✔	✔	✔	✔	9
Abraha et al. 2018 [50]	✔	✔	✔	✔	✔	✔	✔	✔	✔	9
Embafrash and Mekonnen2019 [59]	✔	✔	✔	✔	✔	✔	✔	✔	✔	9
Idowu et al. 2015 [52]	✔	✔	✔	✔	✔	✔	✔	✔	✔	9
Keesara et al. 2018 [57]	✔	✔	✔	X	✔	✔	✔	✔	✔	8
Tegegn et al. 2017 [47]	✔	✔	✔	✔	✔	✔	✔	✔	✔	9
Thindwa et al. 2019[55]	✔	✔	✔	X	✔	✔	X	✔	✔	7
Abraha et al. 2017 [42]	✔	✔ ✔	✔	✔	✔	✔	✔	✔	✔	9
Gejo et al. 2019 [49]	✔	X	✔	✔	✔	✔	✔	✔ ✔	✔	8
Gebremedhin et al. 2018 [44]	✔	✔	✔	✔	✔	✔	✔	✔	✔	9
Mengesha et al. 2015 [48]	✔	✔	✔	✔	✔	✔	✔	✔	✔	9
Berta et al. 2018 [46]	✔	✔	✔	✔	✔	✔	✔	✔	✔	9
Achwoka et al. 2018 [56]	✔	X	✔	X	X	✔	✔	✔	X	5
Abera et al. 2015 [41]	✔	✔	✔	✔	✔	✔	✔	✔	✔	9
Dona et al. 2018 [45]	✔	✔	✔	✔	✔	✔	✔	✔	✔	9
Iliyasu et al. 2018 [53]	✔	✔	✔	✔	✔	✔	✔	✔	✔	9
Chinaeke et al. 2019 [51]	✔	✔	✔	✔	✔	✔	✔	✔	✔	9
Sileo et al. 2015 [58]	✔	✔	✔	✔	✔	✔	✔	✔	✔	9
Bwazi et al. 2014 [54]	✔	✔	✔	X	✔	✔	✔	✔	✔	8
Total	19	17	19	15	18	19	18	19	18	

## Abbreviations

PPFP: Postpartum family planning
ANC: Antenatal care
PNC: Postnatal care
SSA: Sub-Sahara (n)
FP: Family planning
UN: United Nations
USA: United States of America
